# Clinical observation of ultrasound-guided microwave ablation for 1717 benign breast lesions: a multicenter study

**DOI:** 10.1186/s13244-026-02361-x

**Published:** 2026-08-10

**Authors:** Ying Zhou, Yuan Liu, Hang Li, Pei-Yao Wang, Yi-Fan Wang, Ming-Kui Li, Chang-Fu Zheng, Zhi-Feng Xu, Shu-Wei Wang, Hong-Ling Wang, Shu-Rong Wang, Dong Xu

**Affiliations:** 1 Department of Surgery, Hebei Provincial Hospital of Traditional Chinese Medicine, Shijiazhuang, China; 2 Faculty of Applied Sciences, Macao Polytechnic University, Macao, China; 3 Department of Breast Surgery, Affiliated Hospital of Putian University, Putian, China; 4 Department of Diagnostic Ultrasound Imaging and Interventional Therapy, Cancer Hospital of the University of Chinese Academy of Sciences (Zhejiang Cancer Hospital), Hangzhou, China; 5 Department of Ultrasonography, Xiaoshan Hospital, Hangzhou, China; 6 Department of Diagnostic Ultrasound Imaging and Interventional Therapy, Taizhou Cancer Hospital, Wenling, China; 7 Department of General Surgery, Xinkaiyuan Hospital, Xiamen, China; 8 Department of Medical Ultrasound, Yantai Hospital of Shandong Wendeng Orthopaedics and Traumatology, Yantai, China

**Keywords:** Ablation techniques, Benign breast lesion, Ultrasound-guided

## Abstract

**Objectives:**

To evaluate the clinical effectiveness of ultrasound-guided microwave ablation (MWA) for benign breast lesions (BBLs).

**Materials and methods:**

This prospective multicenter study (January 2020 to July 2023) included 1043 patients from seven centers with 1717 biopsy-proven benign breast lesions. The study analyzed contrast-enhanced ultrasound (CEUS)-confirmed ablation outcomes, complications, volume reduction ratio (VRR), cosmetic satisfaction, and time of complete absorption (TCA).

**Results:**

On postoperative day 1, CEUS demonstrated complete non-enhancement in 1713/1717 lesions (99.77%) after the initial ablation; four lesions with residual enhancement underwent immediate supplementary ablation with subsequent CEUS-confirmed non-enhancement. The mean maximum lesion diameter was 1.38 ± 0.25 cm. After a median follow-up of 38.7 months, 1643 out of 1717 lesions (95.69%) were not palpable. The 12-month VRR was 99.98% for lesions ≤ 1.0 cm, 98.20% for lesions 1.0 to 2.0 cm, and 85.43% for lesions ≥ 2.0 cm. VRR was significantly associated with lesion size and procedural parameters, including ablation power and the number of antenna insertions (all *p* < 0.001). Postoperative pain was mild (VAS 0.52 ± 0.43), and no patients discontinued treatment due to pain. Minor complications included fat liquefaction in 3 of 1717 lesions (0.17%) and self-limiting nodularity or fibrosis in 106 lesions (6.2%). No major complications, such as burns, infection, or bleeding, occurred. Cosmetic satisfaction was rated as excellent in 92.4% of patients and good in 7.6%.

**Conclusion:**

Percutaneous MWA is a safe, effective, and minimally invasive treatment for BBLs, providing durable lesion regression and excellent cosmetic outcomes with minimal self-limiting complications.

**Critical relevance statement:**

This multicenter study supports ultrasound-guided microwave ablation as a feasible minimally invasive treatment for benign breast lesions, providing durable long-term regression and favorable cosmetic outcomes while helping reduce procedure-related tissue trauma associated with surgery.

**Key Points:**

- Long-term multicenter evidence for ultrasound-guided microwave ablation of benign breast lesions remains limited, particularly regarding safety, lesion regression, and cosmetic outcomes.
- Microwave ablation achieved 99.77% day-1 contrast-enhanced ultrasound (CEUS) non-enhancement, durable lesion regression, minimal complications, and high cosmetic satisfaction during long-term follow-up.

**Graphical Abstract:**

**Figure:**
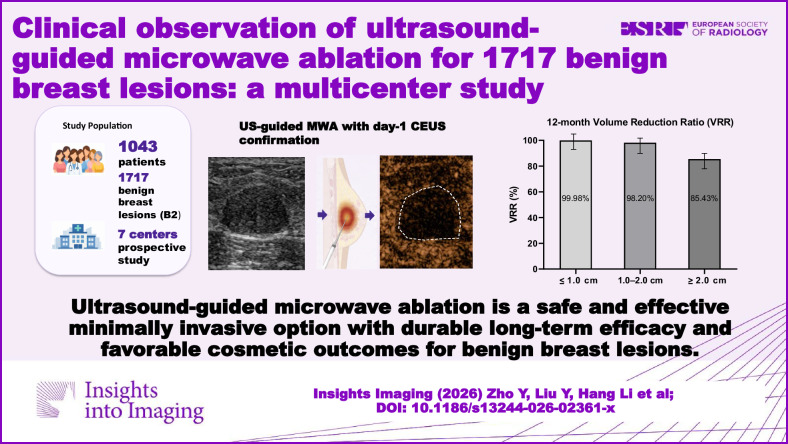

## Introduction

Benign breast lesions (BBLs) account for 60% to 85% of all breast disease [1, 2]. Among lesions requiring biopsy, approximately 80% are confirmed to be benign [3]. The most common histological types are fibroadenoma and mammary adenosis, which typically present clinically as multiple palpable lesions or masses [4–6], resulting from ductal or lobular tissue hyperplasia [7–9]. Conventional management options include regular follow-up, surgical excision, and minimally invasive therapy. While some nodules regress spontaneously, many remain palpable or cause pain, swelling, or nipple discharge [10]. Although most benign lesions can be safely observed, surgery is often performed for symptomatic fibroadenomas, indeterminate imaging findings, or patient anxiety. About 30–50% of such patients opt for excision [8, 9, 11]. While surgical excision offers significant therapeutic efficacy, it is often accompanied by visible scarring, breast contour deformity, nipple displacement, or volume reduction [8], particularly affecting younger women who prioritize cosmetic outcomes. Ultrasound-guided vacuum-assisted biopsy (VAB), a minimally invasive approach, is widely used; however, it similarly carries risks of complications, including hematoma formation, residual nodules, and skin retraction [12–14]. These limitations underscore the need for alternative minimally invasive treatment options that preserve breast aesthetics. Collectively, these shortcomings highlight an unmet need for a scar-sparing, office-based treatment that can reliably eradicate benign lesions while preserving breast cosmesis. Consequently, energy-based ablative techniques—cryotherapy, microwave ablation (MWA), radiofrequency ablation (RFA), laser ablation (LA), and high-intensity focused ultrasound (HIFU)—are increasingly applied for BBLs [15–23]. Compared with other ablation techniques, MWA offers several advantages: higher and more homogeneous intratumoral temperatures, larger ablation zones, shorter ablation times, and lower dependence on tissue electrical conductivity [24, 25]. However, as a relatively new technology, reports on MWA for benign breast nodules remain limited, with few multicenter studies and short follow-up periods [26, 27]. Therefore, we conducted a large, multicenter study to evaluate the safety, efficacy, and long-term outcomes of ultrasound-guided percutaneous MWA for benign breast lesions.

## Materials and methods

### Study design and overview

**Fig. 1:**
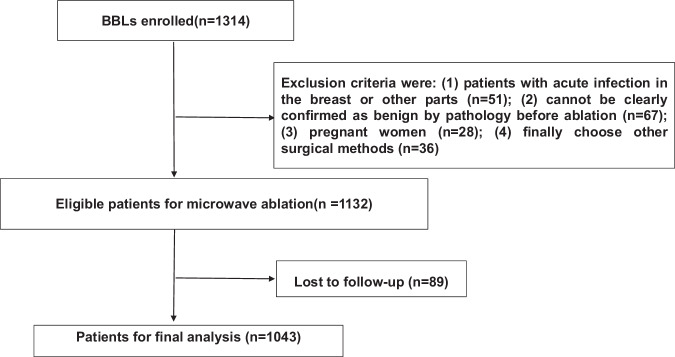
Flowchart of patient enrollment and selection for microwave ablation analysis. A total of 1314 patients with benign breast lesions (BBLs) were initially enrolled. Patients were excluded because of acute infection, inability to confirm benign pathology before ablation, pregnancy, or preference for alternative surgical treatment. After exclusion and follow-up screening, 1043 patients were included in the final analysis

This multicenter prospective study was conducted from January 2020 to July 2023 at seven hospitals across different regions of China. Patients with biopsy-proven benign breast lesions (BBLs) who provided informed consent were consecutively enrolled, and eligibility was not restricted to those refusing surgery; rather, all patients meeting the predefined inclusion criteria were considered eligible. Figure 1 illustrates the screening and enrollment flowchart (number screened, excluded, and included). MWA procedures were performed by four interventional radiologists (IRs) and three surgeons, each with over 5 years of ablation experience. The study was approved by the Medical Ethics Committee of Hebei Provincial Hospital of Traditional Chinese Medicine (No.: 2024-KY-069) and was conducted in compliance with the Declaration of Helsinki.

### Patient enrollment

The following criteria were used to determine inclusion: (1) Only lesions classified as benign B2, as defined by international core biopsy pathology consensus (benign without atypia, e.g., fibroadenomas and adenosis), were included for ablation [28]; (2) Breast lesions identifiable on ultrasound with documented lesion localization; (3) For solitary lesions, a maximum diameter ≥ 1.0 cm was required; for patients with multiple lesions, at least one lesion had to measure ≥ 1.0 cm in maximum diameter. No strict upper size limit was applied; all lesions required core biopsy confirmation of benign B2 pathology; (4) Presence of symptoms (e.g., pain, discharge, discomfort) or patient anxiety regarding lesion growth or malignant potential, prompting a request for treatment despite benign histology; (5) physical condition or cosmetic considerations that caused the patient to refuse other treatments; (6) BI-RADS 4a lesions were included only when core needle biopsy confirmed benign pathology (B2 or lower), radiologic–pathologic concordance was established at multidisciplinary review, and patients expressed a preference to avoid cosmetic damage from surgery. To minimize the risk of false-negative biopsy results, higher-suspicion lesions (BI-RADS 4b and 4c) were excluded, for which surgical excision was recommended; (7) Patients provided written informed consent for ultrasound-guided percutaneous MWA and acknowledged that post-ablation lesion regression is gradual and may vary across individuals.

Exclusion criteria were as follows: (1) B3 lesions with uncertain malignant potential (e.g., phyllodes tumors, atypical ductal hyperplasia, atypical lobular hyperplasia) were explicitly excluded to ensure patient safety and pathological homogeneity; (2) Lesions with BI-RADS ≥ 4b were excluded, for which surgical excision was recommended; (3) Patients with severe coagulation insufficiency; (4) Patients with severe dysfunction of important organs; (5) Patients with acute infection in the breast or other parts; (6) Pregnant and lactating women; (7) Those who have strict requirements on the subsidence time of nodules after ablation; (8) Lesions that could not be adequately visualized on color doppler ultrasound. A consent form was provided to all patients to ensure informed decision-making. None of the patients opted out of the study after signing the consent form.

### Pre-procedure evaluation

Ultrasound (US) was used as the primary imaging modality for baseline evaluation because it is widely available, radiation-free, cost-effective, and well-suited for repeated assessment and follow-up of benign breast lesions. Baseline US was performed to: (1) characterize lesion size, location, margins, depth, and vascularity before ablation; (2) guide core needle biopsy to confirm benign pathology prior to enrollment. As defined by sonographic assessment, BBLs < 2 mm from the skin, areola, or pectoralis major muscle were considered adjacent to these structures. This definition was used to identify lesions requiring additional protective measures (e.g., hydrodissection) rather than to define a therapeutic ablation margin. To calculate baseline lesion volume, three perpendicular diameters were measured on US imaging (a: left–right, b: anterior–posterior, c: superior–inferior) [29], and volume was calculated as: Volume = π/6 × a × b × c.

### US-guided MWA

**Fig. 2:**
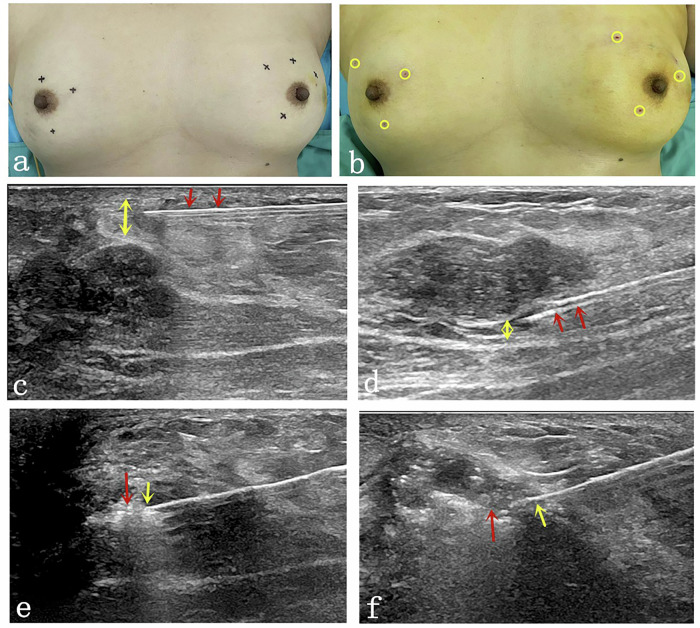
Hydrodissection-assisted ultrasound-guided microwave ablation for bilateral benign breast lesions adjacent to critical structures. **a** Pre-ablation skin appearance and lesion localization. **b** Post-procedural skin appearance demonstrating only minimal puncture entry sites after microwave ablation (yellow circles), with no obvious skin damage or cosmetic deformity. **c** Saline hydrodissection was performed between the lesion and skin to increase the safety distance and reduce thermal injury risk (red arrows: needle; yellow arrow: hydrodissection space). **d** Ultrasound-guided positioning of the microwave antenna tip at the deepest treatment point within the lesion. **e** Formation of the hyperechoic ablation zone during microwave ablation. **f** Stepwise withdrawal of the antenna during ablation to achieve complete lesion coverage. MWA, microwave ablation; US, ultrasound

All participating centers employed the same microwave ablation system (KY-2000, Canyon Medical) and followed a standardized ablation protocol with predefined power settings (typically 30–35 W; up to 40 W in large lesions). Under real-time ultrasound guidance, the antenna was positioned parallel to the long axis of the lesion. Local anesthesia (2% lidocaine and 1% ropivacaine) was administered perilesionally. Hydrodissection was performed with a 23-gauge needle (7 cm; Carrie Co., Ltd) using 10–20 mL of normal saline to create a protective fluid barrier between the lesion and adjacent critical structures (skin, areola, or pectoralis major muscle), thereby minimizing the risk of thermal injury (Fig. 2).

A single microwave antenna was used in all procedures; no multi-antenna approach was used. The antenna was percutaneously inserted into the deepest part of the target lesion and aligned along the long axis under continuous ultrasound guidance. For larger or irregularly shaped lesions, complete coverage was achieved by repositioning the same antenna using the moving-shot technique [10, 30]. During ablation, a hyperechoic zone around the antenna tip was monitored in real time, and the antenna was gradually retracted to extend complete coverage up to the lesion boundary. Unlike malignant tumor ablation, no additional safety margin beyond the lesion boundary was applied, as the objective was complete ablation of the benign nodule while preserving adjacent normal glandular tissue for cosmetic reasons.

Immediate postoperative pain was assessed using a visual analog scale (VAS) and categorized as mild (0–3), moderate (4–6), or severe (7–10) [31]. Postoperative analgesics were not routinely used; patients received ice packing and were observed for 30 min after the procedure.

### CEUS assessment and definition of technical success

Contrast-enhanced ultrasound (CEUS) has been widely used in thermal ablation studies as a surrogate marker of non-viable tissue [32], providing a reliable and non-invasive alternative to post-ablation histological confirmation. In this study, CEUS was performed using a GE LOGIQ E9 scanner (GE Medical Systems & Primary Care Diagnostics) with a 5.0–9.0 MHz transducer, and SonoVue (Bracco Imaging) as the contrast agent. According to the study protocol, CEUS was scheduled as a single examination on postoperative day 1 to assess the ablation zone and confirm technical success; a well-demarcated non-enhancing area was considered indicative of complete ablation of the target benign breast lesion. Technical success was defined as (1) completion of the intended ablation procedure without intraprocedural complications, (2) complete coverage of the target lesion by the hyperechoic zone on real-time ultrasound, and (3) confirmation of non-enhancement on day-1 CEUS. If only minimal peripheral enhancement without nodular or intralesional enhancement was observed on day-1 CEUS, it was interpreted as reactive inflammatory hyperemia rather than residual viable tissue, and no further intervention was performed; routine follow-up was continued. In contrast, if obvious peripheral or intralesional enhancement was considered suggestive of residual viable tissue and prompted immediate supplementary ablation, followed by repeat CEUS on the same day to reconfirm non-enhancement. Apart from this immediate reassessment after supplementary ablation on day 1, subsequent follow-up imaging relied on conventional ultrasound, and CEUS was not performed routinely. Final technical success was determined after completion of any immediate supplementary ablation performed on postoperative day 1.

### Follow-up and imaging evaluation

After the day-1 CEUS assessment, all subsequent follow-up evaluations were performed using conventional ultrasound to monitor lesion regression, VRR, TCA, cosmetic outcomes, and potential complications. Follow-up visits were scheduled at 3 and 6 months after treatment and every 6 months thereafter until complete lesion absorption, after which follow-up was discontinued. For patients with multiple lesions, follow-up continued until complete absorption of all treated lesions. Lesion volume at each follow-up time point (3, 6, and 12 months) was calculated using the same formula as baseline (Volume = π/6 × a × b × c). VRR was calculated as: VRR = (initial volume − follow-up volume) × 100% / initial volume.

Cosmetic outcomes were assessed using a four-point institutional scale (excellent, good, acceptable, poor) [33]. Breast shape, incision scar, and pigmentation were evaluated by physicians based on clinical examination, whereas nipple sensation and areolar sensation were assessed using patient-reported outcomes during follow-up visits. This institutional scale has not undergone international validation; however, its domains were designed with reference to commonly used instruments such as BREAST-Q [34, 35] and the Harvard/NSABP [36] cosmetic scale.

### Statistical analysis

Statistical analyses were performed using SPSS (version 26.0) and GraphPad Prism (version 8.0). Continuous variables are reported as mean ± standard deviation (SD) or median (interquartile range, IQR), as appropriate, and categorical variables as number (percentage). Group comparisons were conducted using one-way ANOVA for normally distributed variables and the Kruskal–Wallis H test for non-normally distributed variables, and the χ² test or Fisher’s exact test for categorical variables. A two-sided *p*-value < 0.05 was considered statistically significant. Binary logistic regression was used to identify factors associated with complete absorption at 12 months (defined as VRR > 95%). Predictors were dichotomized using prespecified cut-offs based on the cohort distribution (maximum lesion diameter 1.0 cm to align with the main size stratification; ablation time 60 s, close to the overall mean of 55.29 s). Odds ratios (ORs) with 95% confidence intervals (CIs) were reported. Firth’s penalized likelihood was applied when complete separation occurred.

## Results

### Baseline characteristics

The study involved 1043 patients with 1717 lesions who underwent US-guided percutaneous MWA and had a minimum follow-up of 12 months. All diagnoses were confirmed by core needle biopsy. The mean age was 37.81 ± 9.99 years (range, 18–56 years). The median follow-up was 38.7 months (range, 12–54 months). The mean maximum lesion diameter was 1.38 ± 0.25 cm (range, 0.2–5.6 cm). Most lesions were palpable before ablation (1452/1717, 84.57%). Lesion locations were parenchymal in 66.5% (1142/1717), adjacent to the pectoralis major muscle in 13.8% (237/1717), near the skin in 10.3% (176/1717), and near the areola in 9.4% (162/1717) (Table 1).

**Table 1:** Baseline characteristics of patients and lesions

Variable	Data
Mean age (years)	37.81 ± 9.99
Breastfeeding history (*n*, %)	
Yes	856 (82.07%)
No	187 (17.93%)
Lesion size category (*n*, %)	
≤ 1 cm	1339 (78.0%)
1.0–2.0 cm	295 (17.2%)
≥ 2 cm	83 (4.8%)
Maximum lesion diameter (cm)	1.38 ± 0.25 (range, 0.2–5.6)
Lesion location (*n*, %)	
In the parenchyma	1142 (66.5%)
Adjacent to the pectoralis	237 (13.8%)
Adjacent to the skin	176 (10.3%)
Adjacent to the areola	162 (9.4%)
Histopathology (*n*, %)	
Fibroadenoma	943 (54.92%)
Fibroepithelial tumor	501 (29.18%)
Adenosis	209 (12.17%)
Hyperplastic nodule	64 (3.73%)
Reasons for ablation (*n*, %)	
Palpable lesions	1452 (84.57%)
Psychological pressure	1361 (79.27%)
BI-RADS 3	816 (47.52%)
BI-RADS 4a	422 (24.58%)
Gradual growth	694 (40.42%)
Family history of breast cancer	129 (7.51%)
Pain	68 (3.96%)
Mean ablation time (s)	55.29 ± 41.53 (range, 7–402)
Mean ablation power (W)	31.12 ± 2.09 (range, 30–40)
Mean ablation insertions (*n*)	1.75 ± 1.13 (range, 1–7)
Median follow-up (months)	38.7 (range, 12–54)

Patient-level variables are reported per patient (*n* = 1043), whereas lesion-related variables are reported per lesion (*n* = 1717)

### Treatment effectiveness

The mean ablation time was 55.29 ± 41.53 s (range, 7–402 s), the mean number of antenna repositionings/insertions was 1.75 ± 1.13 (range, 1–7), and the mean ablation power was 31 ± 2.09 W (range, 30–40 W). Before MWA, lesions appeared as well-circumscribed masses on gray-scale ultrasound. During ablation, a hyperechoic zone formed around the antenna tip, and the lesion margins became ill-defined and heterogeneous (Fig. 2e, f).

**Fig. 3:**
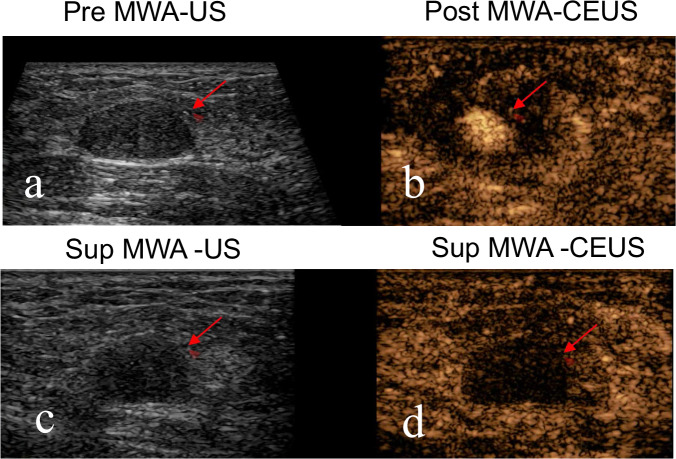
Contrast-enhanced ultrasound assessment before and after supplementary microwave ablation for residual viable tissue. **a** Pre-ablation ultrasound demonstrated a heterogeneous hypoechoic benign breast lesion. **b** Post-ablation CEUS on postoperative day 1 demonstrated residual peripheral and intralesional enhancement suggestive of viable residual tissue, prompting supplementary ablation. **c** Ultrasound after supplementary ablation demonstrated a heterogeneous hyperechoic ablation zone. **d** Repeat CEUS after supplementary ablation confirmed complete non-enhancement of the treated lesion. MWA, microwave ablation; US, ultrasound; CEUS, contrast-enhanced ultrasound; Sup, supplementary

On postoperative day 1, CEUS demonstrated complete non-enhancement in 1713/1717 lesions (99.77%). Four lesions showed obvious peripheral and/or intralesional enhancement, suggestive of residual viable tissue, and therefore underwent immediate supplementary ablation, followed by repeat CEUS on the same day to confirm non-enhancement (Fig. 3). An additional 18 lesions (1.05%) showed minimal peripheral enhancement on day-1 CEUS without nodular or intralesional enhancement. These findings were considered consistent with reactive inflammatory hyperemia; therefore, no additional intervention was performed. All 18 lesions subsequently showed complete disappearance on routine follow-up ultrasound without delayed re-intervention.

### Volume reduction ratio

**Fig. 4:**
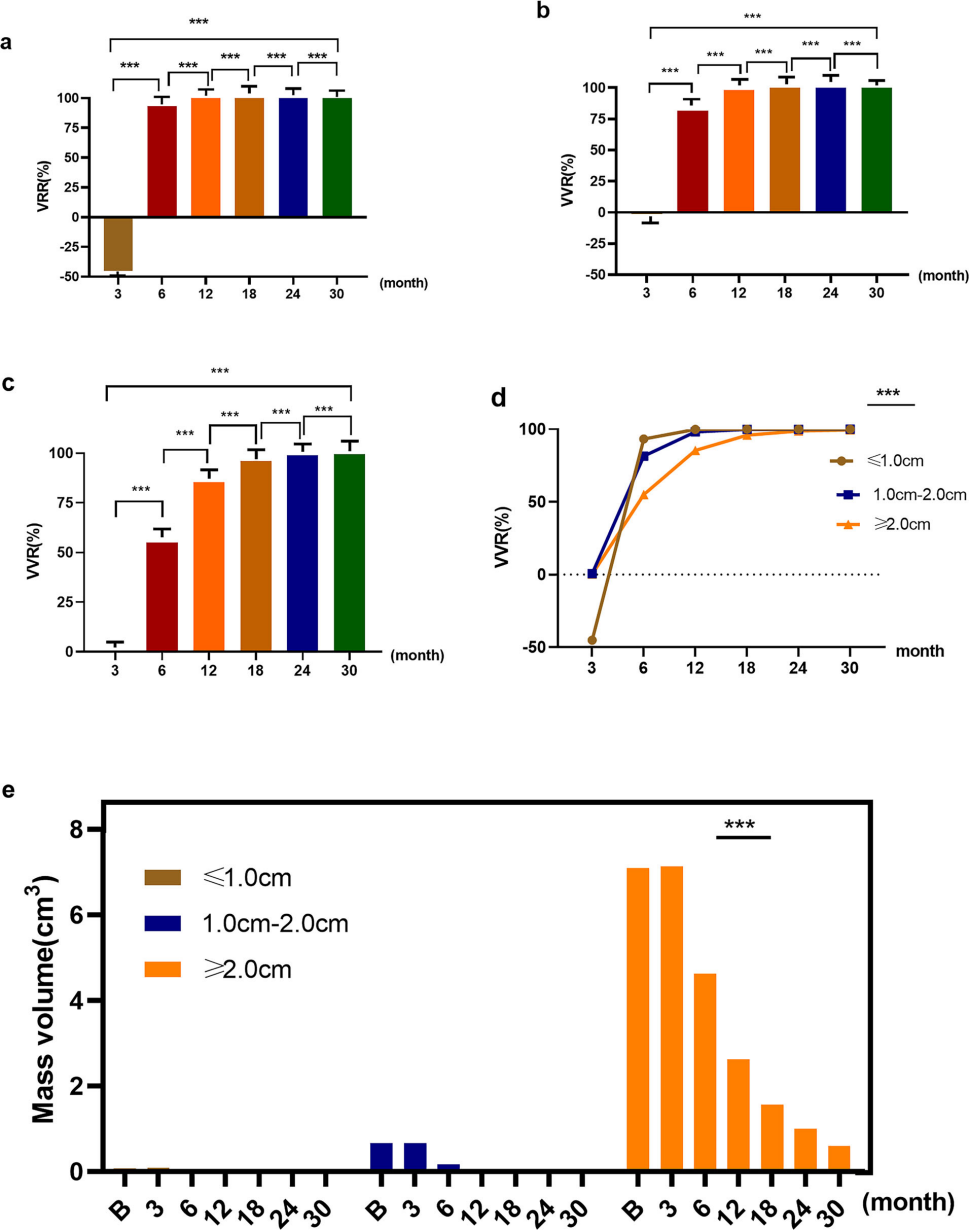
Longitudinal volume reduction ratio and lesion volume changes after microwave ablation stratified by lesion size. **a** Volume reduction ratio (VRR) for lesions ≤ 1.0 cm. **b** VRR for lesions 1.0–2.0 cm. **c** VRR for lesions ≥ 2.0 cm. **d** Longitudinal trends in VRR during follow-up. **e** Longitudinal trends in lesion volume during follow-up. Data are presented as mean ± SD. B indicates pre-ablation baseline. ** *p* < 0.001 (Kruskal–Wallis test)

Lesions were stratified by maximum diameter into three groups: ≤ 1.0 cm, 1.0–2.0 cm, and ≥ 2.0 cm. VRR and lesion volume were assessed at 3, 6, 12, 18, 24, and 30 months after ablation (Table 2 and Fig. 4).

**Table 2:** Longitudinal changes in lesion volume and volume reduction ratio after ultrasound-guided microwave ablation stratified by lesion size

Variable	≤ 1.0 cm (*n* = 1339)	1.0–2.0 cm (*n* = 295)	≥ 2.0 cm (*n* = 83)	*H*	*p*-value
Ablation time (s), median (IQR)	35 (21–50)	107 (100–119)	148 (133–178)	886.728	< 0.001
Ablation power (W), median (IQR)	30 (30–30)	30 (30–35)	35 (35–35)	582.130	< 0.001
Insertion (*n*), median (IQR)	1 (1–1)	3 (3–4)	5 (4–5)	8136.706	< 0.001
Volume (cm^3^), median (range)					
Before ablation	0.07 (0.00–1.66)	0.66 (0.07–2.38)	7.10 (0.37–184.67)	521.634	< 0.001
3 months after ablation	0.09 (0.00–1.77)	0.66 (0.04–2.53)	7.13 (0.33–186.61)	793.705	< 0.001
6 months after ablation	0.00 (0–0.52)	0.17 (0–1.03)	4.63 (0.01–155.37)	645.065	< 0.001
12 months after ablation	0.00 (0–0.17)	0.02 (0–0.17)	2.63 (0.00–119.96)	1057.960	< 0.001
18 months after ablation	0.00 (0–0.00)	0.00 (0–0.04)	1.56 (0–88.93)	1314.103	< 0.001
24 months after ablation	0.00 (0–0.00)	0.00 (0–0.01)	1.00 (0–65.24)	766.178	< 0.001
30 months after ablation	0.00 (0–0.00)	0.00 (0–0.00)	0.60 (0–42.33)	297.723	< 0.001
VRR (%)					
3 months after ablation	−45.08 ± 69.17	0.63 ± 16.93	0.30 ± 9.19	242.273	< 0.001
6 months after ablation	93.36 ± 9.69	81.56 ± 12.32	55.12 ± 14.44	450.915	< 0.001
12 months after ablation	99.98 ± 0.33	98.20 ± 2.56	85.43 ± 11.95	1041.714	< 0.001
18 months after ablation	100.00 ± 0.00	99.91 ± 0.37	95.97 ± 6.76	1304.876	< 0.001
24 months after ablation	100.00 ± 0.00	99.99 ± 0.00	98.84 ± 4.48	764.833	< 0.001
30 months after ablation	100.00 ± 0.00	100.00 ± 0.00	99.54 ± 2.73	297.723	< 0.001

Data are presented as median (range), median (IQR), or mean ± SD, as appropriate. *H* represents the Kruskal–Wallis H statistic

*VRR* volume reduction ratio

For lesions ≤ 1.0 cm, VRR increased rapidly over time (3 months: −45.08 ± 69.17%; 6 months: 93.36 ± 9.69%; 12 months: 99.98 ± 0.33%) and reached 100% thereafter. For lesions 1.0–2.0 cm, VRR was 0.63 ± 16.93% at 3 months, 81.56 ± 12.32% at 6 months, and 98.20 ± 2.56% at 12 months, approaching 100% during longer follow-up. For lesions ≥ 2.0 cm, VRR increased more gradually (3 months: 0.30 ± 9.19%; 6 months: 55.12 ± 14.44%; 12 months: 85.43 ± 11.95%), with continued improvement up to 30 months (99.54 ± 2.73%). Negative VRR values were observed at early follow-up, particularly in lesions ≤ 1.0 cm. This likely reflects transient enlargement of the ablation zone relative to the pre-ablation lesion volume due to perilesional thermal spread and edema.

Overall, VRR increased progressively over time across all size groups, with the most pronounced reduction occurring within the first 6 months after ablation. Between-group differences in VRR and lesion volume were significant at each follow-up time point (all *p* < 0.001; Fig. 4d, e). The slower regression in larger lesions is consistent with the longer time required for resorption and remodeling of a larger ablation zone.

### Factors associated with TCA and 12-month VRR

At 6 months, complete absorption (CA) was observed in 808/1339 (60.3%) lesions ≤ 1.0 cm, 52/295 (17.6%) lesions 1.0–2.0 cm, and 1/83 (1.2%) lesions ≥ 2.0 cm. At 12 months, CA was observed in 1338/1339 (99.9%), 251/295 (85.1%), and 13/83 (15.7%) lesions, respectively (Table 3; all *p* < 0.001).

**Table 3:** Complete and partial absorption rates of benign breast lesions at 6 and 12 months after microwave ablation stratified by lesion size

Time	Variable	≤ 1.0 cm (*n* = 1339)	1.0–2.0 cm (*n* = 295)	≥ 2.0 cm (*n* = 83)	χ²	*p*-value
6 months	PA	531 (39.7%)	243 (82.4%)	82 (98.8%)	260.003	< 0.001
	CA	808 (60.3%)	52 (17.6%)	1 (1.2%)		
12 months	PA	1 (0.1%)	44 (14.9%)	70 (84.3%)	926.484	< 0.001
	CA	1338 (99.9%)	251 (85.1%)	13 (15.7%)		

*PA* partial absorption, *CA* complete absorption, defined as VRR > 95% at the corresponding follow-up time point

**Fig. 5:**
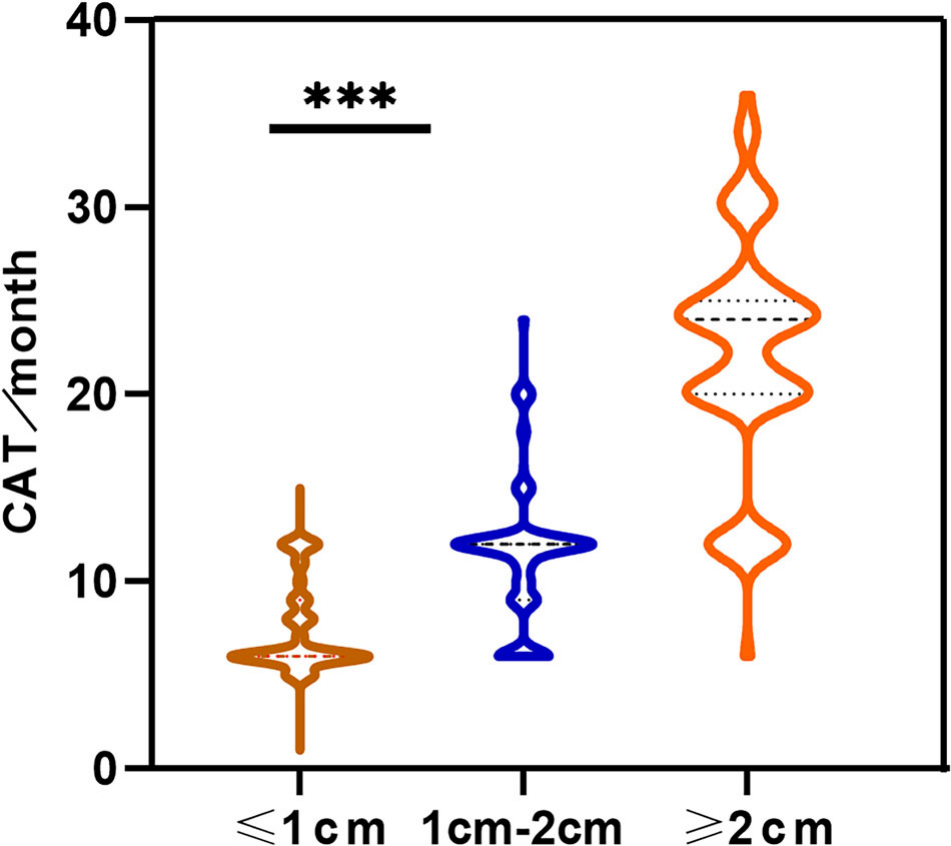
Time to complete absorption after microwave ablation stratified by lesion size. Time to complete absorption (TCA) was compared among lesions ≤ 1.0 cm, 1.0–2.0 cm, and ≥ 2.0 cm after microwave ablation. Larger lesions demonstrated significantly prolonged absorption time during follow-up. *** *p* < 0.001

The median time to complete absorption (TCA) was 6 months (IQR, 6–9) for lesions ≤ 1.0 cm, 12 months (IQR, 9–12) for lesions 1.0–2.0 cm, and 24 months (IQR, 20–25) for lesions ≥ 2.0 cm (*p* < 0.001; Fig. 5).

In univariate logistic regression analyses, maximum lesion diameter, ablation time, ablation power, and the number of antenna repositionings/insertions were significantly associated with complete absorption at 12 months (Table 4; all *p* < 0.001).

**Table 4:** Univariable logistic regression analysis identifying factors associated with complete absorption of benign breast lesions at 12 months after microwave ablation

Variable	Category	Distribution *n* (%)	CA	PA	OR	95% CI	*p*-value
Maximum lesion diameter (cm)	≤ 1.0 (Ref.)	1339 (77.98%)	1338	1			
	> 1.0	378 (22.02%)	264	114	< 0.01	0.00023–0.012	< 0.001
Ablation time (s)	≤ 60 (Ref.)	1154 (67.21%)	1154	0			
	> 60	563 (32.79%)	448	115	< 0.01	0.00012–0.027	< 0.001
Ablation power (W)	≤ 30 (Ref.)	1407 (81.95%)	1400	7			
	> 30	310 (18.05%)	202	108	< 0.01	0.004–0.020	< 0.001
Insertions (*n*)	≤ 2 (Ref.)	1346 (78.39%)	1345	1			
	> 2	371 (21.61%)	257	114	< 0.01	0.00021–0.012	< 0.001

ORs compare each listed category with the corresponding reference category (Ref.). Firth’s penalized likelihood was applied when complete separation occurred

*CA* complete absorption, *PA* partial absorption, *OR* odds ratio, *CI* confidence interval, *VRR* volume reduction ratio

### Cosmetic outcomes and complications

Minor complications included fat liquefaction in 3/1717 lesions (0.17%), which resolved spontaneously within 3 weeks, and palpable nodularity or fibrosis in 106/1717 lesions (6.2%). At a median follow-up of 38.7 months, 1643/1717 lesions (95.69%) were no longer palpable on clinical examination. Persistent firmness at the treated site was reported in 106/1717 lesions (6.2%), whereas the remaining lesions showed gradual softening over time. Postoperative pain intensity was assessed using a visual analog scale (VAS). Immediate postoperative pain was generally mild (VAS 0.52 ± 0.43), self-limiting and not requiring analgesics, and no patients discontinued treatment because of pain. No major complications, including skin or muscle burns, infection, nerve injury, or active bleeding, were observed. Very mild, transient post-procedural events (e.g., limited edema, ecchymosis, minor erythema, small self-limited hematomas, or early induration) could occur after percutaneous ablation; however, they were not prospectively and systematically captured as reportable complications in this multicenter registry because they were short-lived, required no medical intervention, and resolved spontaneously. Regarding cosmetic satisfaction, 964/1043 patients (92.4%) rated the outcome as excellent and 79/1043 (7.6%) as good.

## Discussion

This multicenter prospective study evaluated ultrasound-guided percutaneous MWA for benign breast lesions in 1043 patients with 1717 lesions. At a median follow-up of 38.7 months, the majority of lesions treated became non-palpable, with faster regression in smaller lesions. Overall, MWA was associated with minimal pain and no major complications, supporting its role as a minimally invasive option for appropriately selected benign lesions.

With the increasing emphasis on minimally invasive methods in modern clinical practice, treatment decisions for benign breast lesions are increasingly focusing on preserving function and cosmetic outcomes, in addition to efficacy [8]. Compared with minimally invasive techniques, surgical excision is associated with greater tissue disruption, visible scarring, and potential contour deformity, which may be unacceptable for many patients. Concerns regarding complications [12, 37, 38] and the need for larger incisions in selected cases [18] further reduce the acceptability of surgery. Accordingly, minimally invasive approaches such as ultrasound-guided vacuum-assisted resection (US-VAE) and US-guided ablation have received considerable attention [39]. In the treatment of BBLs with minimally invasive techniques, cryoablation, radiofrequency ablation, microwave ablation, high-intensity focused ultrasound (HIFU), and laser ablation (LA) are increasingly important [17, 21]. MWA induces tissue necrosis through thermal coagulation and protein denaturation [40, 41]. The advantages of microwave energy include enhanced intratumoral heating, faster ablation times, and more stable energy delivery [24, 25, 42]. Ultrasound-guided microwave ablation has been reported as an effective treatment for BBLs, with favorable cosmetic outcomes and a scar-sparing profile, providing an alternative to surgery [43–45].

Previous prospective studies have provided important evidence supporting US-guided MWA for benign breast lesions, although most were limited by smaller sample sizes, single-center designs, or shorter follow-up. Zhang et al [27] reported 314 patients with 725 lesions and found that 679/694 lesions showed no enhancement immediately after MWA, while 15 lesions larger than 2.5 cm required supplementary ablation because of peripheral enhancement; the disappearance rate reached 93.0% at 18 months, with no major complications. These findings are consistent with our CEUS-based management strategy and gradual lesion regression after MWA. Yang et al [26] further demonstrated the feasibility of US-guided MWA in a prospective multicenter cohort of 755 benign breast lesions. Our study extends this evidence by including 1717 lesions from seven centers, applying stricter pathology-based eligibility criteria, and providing longer follow-up with size-stratified absorption outcomes. The slower regression observed in our ≥ 2.0 cm subgroup is also consistent with Cui et al [46], who reported a 12-month VRR of 80.2 ± 13.1% in 104 biopsy-proven lesions ≥ 2 cm. In our study, lesions ≥ 2.0 cm had a 12-month VRR of 85.43 ± 11.95% and continued to shrink to 99.54 ± 2.73% at 30 months. These comparisons suggest that MWA provides durable lesion regression, but larger lesions require longer follow-up and more cautious counseling regarding delayed absorption and persistent palpability. Compared with previous studies, the strengths of our study include its larger multicenter cohort, longer follow-up, standardized protocol, lesion-level complication reporting, and detailed size-stratified analysis of VRR and TCA.

In comparison with other thermal ablation techniques, the durable lesion control observed in our cohort during a median follow-up of 38.7 months is consistent with prior reports of RFA and LA, which typically demonstrate local control rates above 90% at 1–2 years [17]. However, MWA achieves faster intratumoral heating and larger ablation volumes, which may contribute to the high initial complete non-enhancement rates on day-1 CEUS (99.77%) and favorable long-term absorption observed in our cohort. With regard to safety, no major complications occurred, while minor events such as palpable nodularity (6.2%) and fat liquefaction (0.17%) were observed; these were self-limited, did not compromise cosmetic outcomes, and are comparable to or lower than complication rates reported for vacuum-assisted excision (bleeding and hematomas up to 5–10%) [12–14], cryoablation (skin changes or residual nodularity ~5%) [19, 20], and other minimally invasive ablation techniques [18, 21]. From an economic perspective, MWA can be performed under local anesthesia in an outpatient setting, avoiding operating room costs and prolonged recovery times associated with surgical excision. Finally, our median follow-up approaching 3 years provides supportive evidence for the durability of MWA, which compares favorably with the limited long-term data available for other ablation modalities.

Although technical success was achieved, lesion size substantially influenced post-ablation remodeling dynamics. Larger lesions required longer follow-up and more individualized counseling because delayed absorption and persistent firmness could still occur despite progressive regression over time. Therefore, microwave ablation appears particularly suitable for small-to-moderate benign lesions, whereas lesions exceeding approximately 3–4 cm (noting that the maximum lesion diameter in our cohort was 5.6 cm) should be approached more cautiously, with individualized counseling regarding the likelihood of delayed shrinkage and potential persistent firmness/palpability. For larger or irregular lesions, a size-adapted approach—including planned antenna repositioning using the moving-shot technique, tailored power and ablation duration to ensure complete coverage, and closer follow-up—may help optimize regression while preserving adjacent normal glandular tissue for cosmetic reasons.

### Practical considerations for clinical application

Several technical aspects merit emphasis for optimal clinical implementation of MWA in benign breast lesions. Comprehensive pre-procedural ultrasound evaluation and continuous real-time ultrasound guidance throughout the procedure are essential. For lesions adjacent to the skin, areola, or pectoralis major muscle, hydrodissection should be routinely employed to minimize the risk of thermal injury. Unlike malignant tumor ablation, benign breast lesion ablation does not require an oncologic safety margin beyond the lesion boundary. For lesions adjacent to critical structures, thermal protection mainly relied on hydrodissection, continuous real-time ultrasound monitoring, and carefully controlled ablation settings, which may explain the absence of major thermal injury in our cohort. In our experience, power settings of 30–35 W (increased to up to 40 W for large lesions) provide effective ablation while preserving adjacent normal glandular tissue and maintaining favorable cosmetic outcomes. Recent multidisciplinary expert consensus recommendations have further highlighted the expanding role of ultrasound-guided thermal ablation in breast diseases, emphasizing its safety profile, technical feasibility, and minimally invasive advantages, which are consistent with the findings of the present study [47].

### Regulatory considerations

Regulatory approval for microwave ablation in benign breast lesions varies across countries, and in some regions, its use remains limited to research settings. This heterogeneity in regulatory status may affect the immediate generalizability of our findings and underscores the need for further multicenter evidence, long-term safety data, and cost-effectiveness analyses.

### Limitations

Limitations include the need for longer follow-up and operator-/center-stratified analyses, imaging-based endpoints without histologic confirmation (day-1 CEUS non-enhancement and ultrasound disappearance), and potential inter-center variability in pathological terminology despite exclusion of B3 lesions. Although BI-RADS 4a lesions required benign biopsy with radiologic–pathologic concordance, sampling error remains possible. Larger lesions regressed more slowly and were more likely to remain palpable, warranting targeted counseling and size-adapted protocols. Cosmetic outcomes were assessed using a non-validated institutional scale without validated patient-reported measures, and very mild transient post-procedural events were not systematically captured.

## Conclusion

In summary, ultrasound-guided MWA represents an effective minimally invasive option for selected benign breast lesions, enabling precise lesion targeting and real-time monitoring. Ultrasound-guided ablation may reduce procedure-related psychological stress and unnecessary tissue trauma. Future research should explore the cost-effectiveness of MWA compared with surgical excision, particularly given that MWA can be performed under local anesthesia in an outpatient setting, potentially avoiding operating room costs and shortening recovery time.

## Data Availability

All data supporting the findings of this study are included in the manuscript. Due to restrictions related to patient privacy and multicenter data governance, the underlying datasets are not publicly available. De-identified data may be made available from the corresponding author upon reasonable request and with approval from all participating centers.

## Abbreviations

AT: Ablation time
BBLs: Benign breast lesions
BI-RADS: Breast Imaging Recording and Data System
CA: Complete absorption
CEUS: Contrast-enhanced US
MWA: Microwave ablation
PA: Partial absorption
TCA: Time of complete absorption
US: Ultrasound
VRR: Volume reduction ratio
